# A Patient Registry to Improve Patient Safety: Recording General Neurosurgery Complications

**DOI:** 10.1371/journal.pone.0163154

**Published:** 2016-09-26

**Authors:** Johannes Sarnthein, Lennart Stieglitz, Pierre-Alain Clavien, Luca Regli

**Affiliations:** 1 Neurosurgery Department, Zurich University Hospital, Zurich, Switzerland; 2 University of Zurich, Zurich, Switzerland; 3 Zurich Neuroscience Center, ETHZ, Zurich, Switzerland; 4 Surgery Department, Zurich University Hospital, Zurich, Switzerland; Universita degli Studi di Palermo, ITALY

## Abstract

**Background:**

To improve the transparency of the local health care system, treatment cost was recently referenced to disease related groups. Treatment quality must be legally documented in a patient registry, in particular for the highly specialized treatments provided by neurosurgery departments.

**Methods:**

In 2013 we have installed a patient registry focused on cranial neurosurgery. Surgeries are characterized by indication, treatment, location and other specific neurosurgical parameters. Preoperative state and postoperative outcome are recorded prospectively using neurological and sociological scales. Complications are graded by their severity in a therapy-oriented complication score system (Clavien-Dindo-Grading system, CDG). Results are presented at the monthly clinical staff meeting.

**Results:**

Data acquisition compatible with the clinic workflow permitted to include all eligible patients into the registry. Until December 2015, we have registered 2880 patients that were treated in 3959 surgeries and 8528 consultations. Since the registry is fully operational (August 2014), we have registered 325 complications on 1341 patient discharge forms (24%). In 64% of these complications, no or only pharmacological treatment was required. At discharge, there was a clear correlation of the severity of the complication and the Karnofsky Performance Status (KPS, ρ = -0.3, slope -6 KPS percentage points per increment of CDG) and the length of stay (ρ = 0.4, slope 1.5 days per increment of CDG).

**Conclusions:**

While the therapy-oriented complication scores correlate reasonably well with outcome and length of stay, they do not account for new deficits that cannot be treated. Outcome grading and complication severity grading thus serve a complimentary purpose. Overall, the registry serves to streamline and to complete information flow in the clinic, to identify complication rates and trends early for the internal quality monitoring and communication with patients. Conversely, the registry influences clinical practice in that it demands rigorous documentation and standard operating procedures.

## 1. Introduction

Neurosurgery provides highly specialised treatments to patients. While the number of patients treated is small compared to other medical disciplines, it is steadily growing with the aging of the population in industrialised countries. To improve the cost transparency of the local health care system, treatment cost was recently referenced to disease related groups (DRG). To define a valid case mix index (CMI), patient status at admission must be well documented. Concurrently, treatment quality must be closely monitored to provide transparency between health care providers with respect to the clinical outcome and the complications during the treatment process [1–3].

For a standardized comparison of the patient’s clinical status, a number of clinical scales are well accepted. While many scales were validated in other fields of medicine, they are widely used also in neurosurgery. The definition of a neurosurgical indication and a neurosurgical intervention is more difficult. Local health care authorities use the International Classification of Diseases for indications (ICD-10)[4] and a national adaptation of the International Classification of Diseases Clinical Modification for interventions (ICD-9-CM)[5]. However, these codes are too course-meshed in neurosurgery.

Complications were defined as any deviation from the normal preoperative course. Sequelae and failure to cure were not included in the definition of complications. To score the severity of a clinical complication, several scales have been proposed and applied in neurosurgery [6–8]. Somewhat earlier, a grading scheme based on the therapeutic consequences was proposed in general surgery and subsequently validated in a large number of cases (Clavien Dindo classification system CDG, Table 1) [9–12].

**Table 1:** Classification systems for the severity of a complication. We compare numbers from our study (Zurich), Milano [25] and Buenos Aires [8]. Clavien Dindo classification system CDG [12]; Landriel scheme [8].

Hospital		Zurich		Milano		Buenos Aires			
Cohort size		1341		1008		1190			
Number of complications registered		325	24%	228	23%	167	14%		
CDG	Definition							Landriel	Definition
Grade 1+2			67%	139	61%	53	32%	Grade 1	Any non-life threatening deviation from normal postoperative course, not requiring invasive treatment
Grade 1	Any deviation from the normal postoperative course without the need for pharmacological treatment or surgical, endoscopic, and radiological interventions. Allowed therapeutic regimens are drugs as antiemetics, antipyretics, analgetics, diuretics, electrolytes, and physiotherapy. This grade also includes wound infections opened at the bedside.	72	22%						
Grade 2	Requiring pharmacological treatment with drugs other than such allowed for grade I complications. Blood transfusions and total parenteral nutrition are also included.	145	45%						
Grade 3	Requiring surgical, endoscopic, or radiological intervention.		25%	63	28%	43	26%	Grade 2	Complication requiring invasive treatment such as surgical, endoscopic, or endovascular interventions
Grade 3a	Intervention not under general anesthesia.	14	4%						
Grade 3b	Intervention under general anaesthesia.	67	21%						
Grade 4	Life-threatening complication (including CNS complications) requiring IC/ICU management.		5%	20	9%	57	34%	Grade 3	Life threatening complication requiring management in ICU
Grade 4a	Single-organ dysfunction (including dialysis).	15	5%						
Grade 4b	Multiorgan dysfunction.	1	0%						
Grade 5	Death of a patient within 30 days after surgery.	11	3%	6	3%	1	1%	Grade 4	Complication resulting in death

To monitor treatment quality, we have implemented a patient registry containing all patients treated by members of our department since 2013. As opposed to clinical studies, which provide evidence from controlled experiments, a patient registry provides medical evidence derived from routine clinical practice[13, 14]. Existing patient registries either do not reflect our case mix [15–17] or do not comply with our local requirements [18]. An attempt to establish a national neurosurgery registry for Switzerland failed and, to our knowledge, nothing comparable exists in German or Austrian neurosurgery. We therefore resorted to programming an in-house patient registry. We selected applicable scales and designed a catalogue of indications and a catalogue of interventions, which reflect the case mix of our neurosurgery department that is specialised on cranial indications.

We describe here the content and structure of our patient registry along the Standards for Quality Improvement Reporting Excellence (SQUIRE) [19] with the aim of transparent in-house quality monitoring, communication with patients, and also to facilitate benchmarking with other neurosurgery health care providers.

## 2. Methods

### 2.1. Context

The patient registry was designed to fulfil three main purposes, the most important of which is quality monitoring as it is used for communication within the clinic, with external partners, and with patients. Second, the administrative staff uses the registry to monitor the completeness of the patient records. Third, the registry provides an overview over the data available for research projects. The combination of these purposes ensures timely, complete, and accurate data registration.

### 2.2. Intervention

The intervention described here is defined as implementing and maintaining a patient registry and reporting to the department. The registry records data of all patients, which were operated on by members of our neurosurgery department since 2013. Data acquisition to the registry is open-ended. We present here data from the interval 2013–2015.

Typically, a patient is first entered into the registry by the secretary of the surgery theatre. The patient number is scanned from the hospital electronic patient record (KIS) and patient name and birth date are entered manually. A surgery case report form is printed out (sCRF, S1 Forms). Checklists are completed before surgery [20]. After surgery, the surgeon marks the indication and the intervention in the respective catalogue as well as the anatomical localisation. The indication catalogue contains one item to state whether the surgery was necessary because of a complication. The surgeon also marks whether the patient was operated on for the first time and whether the surgery had to be rescheduled for medical or administrative reasons. For tumour surgeries the surgeon assesses the extent of resection. The team responsible for intraoperative neurophysiological monitoring (IONM) marks the modalities applied. The administrative staff marks the medical devices used, the times of the surgery, the laboratories where biological material was sent to, and enters all information on the sCRF into the electronic patient registry.

After surgery, the patient is transferred to the intensive care unit (ICU) and from there to the ward. The secretary of the ward prints out an admission case report form and a discharge case report form (aCRF and dCRF, S1 Forms), labels them with stickers from the KIS and makes them available to the physicians at the ward. Children < 15 y are transferred to the children’s hospital and registered there.

### 2.3. Measures

Clinical status at admission is rated on the aCRF in a set of scales comprising the Karnofsky Performance Status Scale (KPS), the modified Rankin Scale (mRS), the Glasgow Coma Scale (GCS), the National Institute of Health Stroke Scale (NIHSS), the Montreal Cognitive Assessment (MoCA). The aCRF also contains questions on the patients’ social status, their employment and educational level from a set provided by the local health care authorities [21]. If a hospital admission became necessary because of a complication of a previous treatment, this is recorded on the aCRF in a dedicated matrix.

Clinical status discharge is rated on the dCRF, which contains the same scales as the aCRF. Only the Glasgow Outcome Score (GOS) is applied instead of the GCS. The dCRF also contains a histopathology catalogue of the most common tumour entities. For a follow-up visit the surgeon’s secretary prints out a follow-up case report form (fCRF, S1 Forms) with scales as in the dCRF. On the fCRF also the employment status is marked. The aCRF, dCRF and fCRF are collected by the data typist and entered into the electronic patient registry.

If a complication occurred, it is marked on the CRF in a catalogue of the most frequent adverse events (AE). Any deviation from the pre-operative status and normal postoperative course is considered a complication: a new motor deficit or a wound infection is counted as a surgical complication; a first time epileptic seizure is also counted as a surgical complication; reoccurring seizures are counted as medical complications caused by inadequate drug dosage; similarly a urinary tract infection. The severity of the complication is rated using the Clavien Dindo classification system (CDG, Table 1) [9–11]. The physician also enters the date of occurrence of the complication and/or the interval in relation to the surgery date. This prevents multiple counting of the same event and also classifies the complication as a transient condition or as a permanent deficit. A deficit is defined as “permanent”, if it persists at the time of the follow-up visit. If the deficit has ceased at the next follow-up visit, its status is changed to “transient”.

The resources required exclusively for maintaining the patient registry consist of the data typist (part-time employed 1 day per week) and the data manager (JS, devoting about 1 day per week to the registry). The surgeons, the secretary of the operating theatre and the physicians on the ward need less than 5 minutes to fill out one CRF and a little more time, if information has to be acquired from the electronic patient records. The workload of the other administrative staff has not been changed by the registry, because other data bases were replaced.

To assure timely completion of the CRFs, we build on the scheme that monitors the status of the report letters to external partners. The administrative staff sets a flag in the electronic patient registry as soon as a surgery report is signed by the surgeon and a discharge report is signed by the treating physician of the ward. In this way the state of the patient flow through the clinic is documented for each patient. If a CRF is missing, this is communicated to the physician in charge on the ward.

To assure the accuracy of the data, each paper CRF that was completed by the physician on the ward is then controlled by the responsible senior physician. If a correction turns out to be necessary, also the CRF in the electronic patient registry is corrected. All patients with complications are listed on a data analysis template in the electronic patient registry (S1 Table), which is regularly discussed in the monthly staff meeting of the neurosurgery department. Here the treating physicians provide the context for the complication. Some cases triggered controversial discussions regarding the severity of the complication. If necessary, data entries are corrected. Since S1 Table also states the number of missing dCRF, both the completeness and correctness of the data can be assessed and improved.

The electronic patient registry was implemented in a relational database in filemaker^®^ (www.filemaker.com). The database was custom designed and programmed by the data manager (JS) with external support by www.hyperdots.ch. Several templates and scripts aid in controlling completeness of the data acquisition. Data safety is assured by a hospital file server and user profiles with different levels of authorisation. During the first year, the patient registry was run on the software provided by the local clinical trials centre (www.secutrial.com). The handling of this web-based software proved to be too tedious and too slow to be acceptable for the administrative staff. Furthermore, data could only be exported to CSV files without data analysis functions. We therefore ported the electronic implementation to filemaker^®^ with the beginning of the second year (2014) of the patient registry.

In research projects on some selected patient groups, clinical information is extracted from the electronic patient records (KIS) and entered in the CRF of the electronic patient registry. The diagnosis groups are neurooncology, neurovascular diseases, spinal neurosurgery, cerebrospinal fluid disorders, and trauma. In the registry the data remains readily available for the department also after completion of the research projects.

### 2.4. Analysis

To describe variation within the data, we present percentages together with 95% confidence intervals (CI) based on the binomial distribution. We used non-parametric statistical methods for hypothesis testing and the Bonferroni correction for multiple comparisons. The analysis was performed with custom scripts in MATLAB^®^ (www.mathworks.com). Statistical significance was established at p<0.05. To reduce variation within the data concerning specific aspects of the patient registry, we selected time intervals from the time on, when the data acquisition of the specific aspect was fully operational.

### 2.5. Ethical considerations

The scientific workup was approved upfront by the local ethics review board (Kantonale Ethikkommission KEK-ZH 2012–0244) and it was registered internationally at clinicaltrials.gov (NCT01628406). The authors report no relevant conflicts of interest.

## 3. Results

### 3.1. Completion of CRFs over time

**Fig 1:**
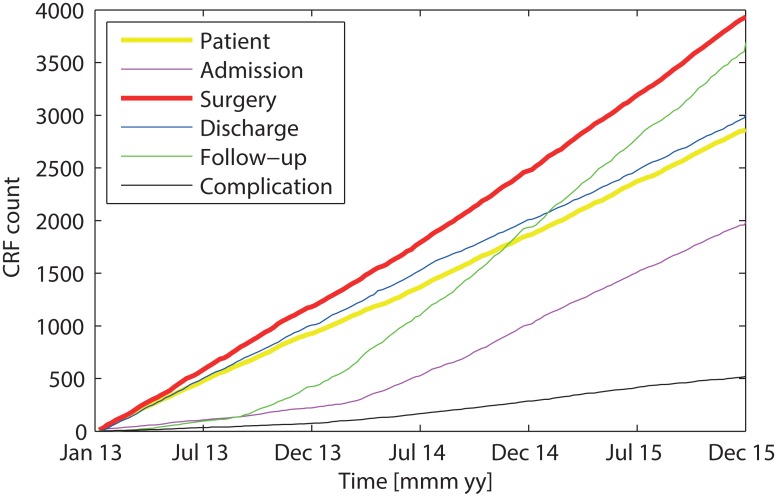
Cumulative sum of case report forms (CRFs) for patients, admissions, surgeries, discharges and follow-ups. Complications registered at discharge (black line).

Fig 1 shows the number of CRFs in the patient registry since its inception. The inclusion of surgeries (sCRF) has proceeded at a regular rate starting from 2014. The number of discharges (dCRF) increases also linearly, albeit at a somewhat lower rate. The lower rate reflects the fact that some patients are operated on several times before discharge and not all patients are discharged from the ward of our department. Registering the surgical interventions and the outcome at discharge was the primary goal during the first months of the implementation of the patient registry. Follow-up visits were registered on a regular basis starting only from 2014 on after changing to filemaker^®^. The slope of the follow-up curve exceeds that of the discharge curve, because several patients appear at several follow-ups. Admissions (aCRF) were included starting summer 2014 and increase in parallel with the discharge curve. The small numbers of admissions and follow-ups before the regular registration were entered from the electronic patient records (KIS) for research projects on select patient groups. From August 2014 on, all dCRF were completed with at least a KPS score. Until the end of 2015 we have registered 2880 patients that were treated in 3959 surgeries and 8528 consultations.

### 3.2. Patient and surgery characteristics

**Fig 2:**
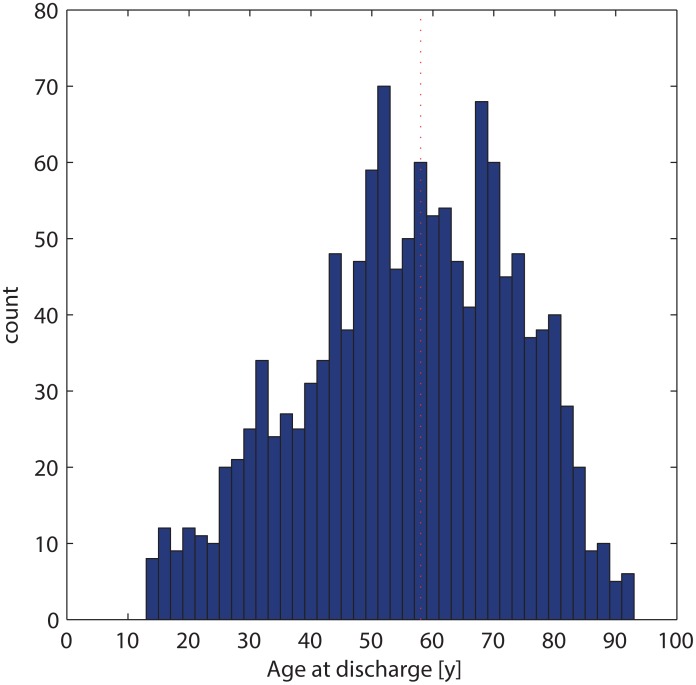
Patient age at discharge. Median 58 y (red line). Children < 15 y were transferred to the children’s hospital and registered there.

At discharge, the age distribution was smoothly distributed around the median (58 y, Fig 2). There were one or more surgeries preceding the discharge and the main indications were neurooncology (34%), neurovascular disease (14%), spinal neurosurgery (14%), trauma (12%), cerebrospinal fluid disorder (10%), other (8%), and complications as a separate category of surgical indication (7%, CDG>2). The percentage of spinal interventions is relatively small, which underlines the cranial focus of our department.

When counting surgeries, there were 256/2041 = 13% CI [11% 14%] surgeries indicated because of a complication since August 2014. This includes complications also from interventions in other hospitals or departments.

### 3.3. Evolution of complications at discharge

Fig 1 shows the rate of registered complications as a black line. Complications were registered in the dCRF on a regular basis starting only from August 2014. A small number of earlier complications were entered from the electronic patient records (KIS) for research projects on select patient groups. The variability in complication rate reflects several factors, among them the number of discharges in the respective month. Another factor is the completeness of registration, which was low for some months. Starting from August 2014, the average rate of complication of any grade at discharge was 325/1341 cases (24% CI [22% 27%], where any deviation from the normal clinical course was registered, be it a surgical or a medical complication. Further complications, like surgical site infections (SSI), were additionally registered after discharge at follow-up visits.

### 3.4. Complications and KPS at discharge

**Fig 3:**
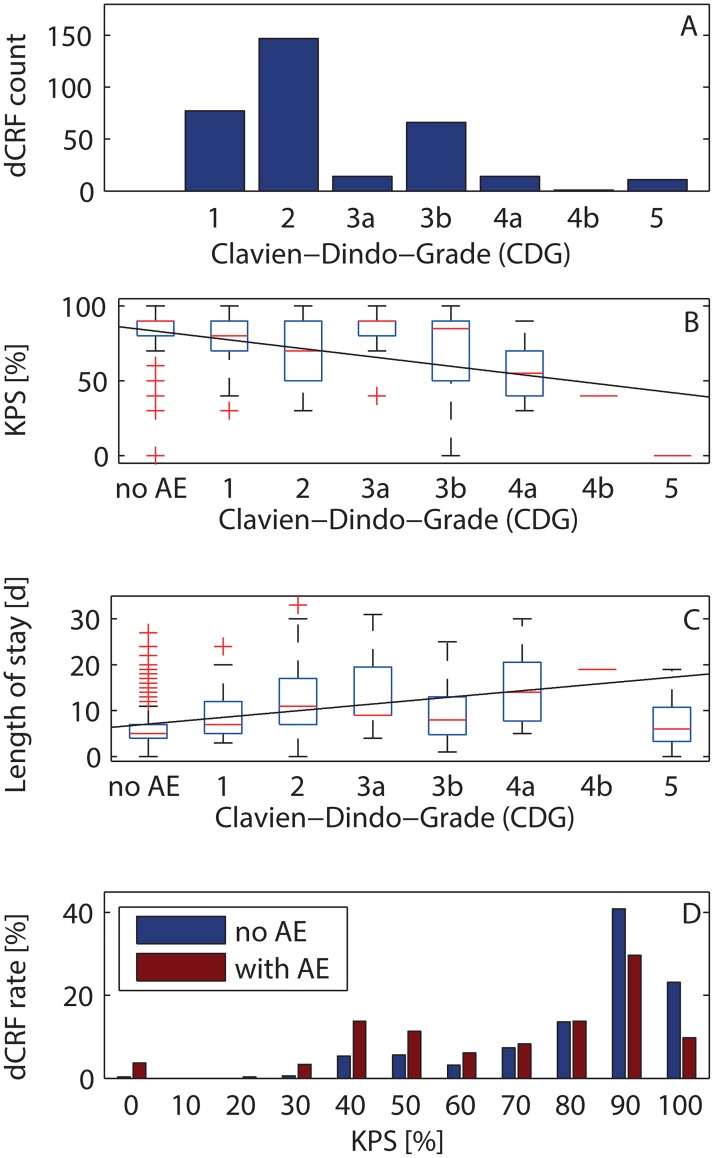
Complications at discharge. (A) Distribution of severity in the Clavien Dindo classification system CDG. (B) Karnofsky Performances Status Scale (KPS) as a function of severity of the complications (ρ = -0.3, slope -6 KPS percentage points per increment of CDG). (C) Duration of hospital stay after surgery (ρ = 0.4, slope 1.5 days per increment of CDG). (D) Distribution of KPS for patients with and without complications (adverse event AE).

Fig 3A shows the distribution of the 325 complications registered at discharge since August 2014. The majority of complications (217/325 = 67% CI [61% 72%]) were treated without invasive treatment (CDG 1 and CDG 2). Eleven patients died within 30 days of surgery (CDG 5). CDG 1 was marked in 72 (22% CI [18% 27%]) patients, including those with a focal neurological deficit, which was not treated and improved in some patients after discharge with the passage of time. An additional surgical intervention under general anaesthesia was required in 67 (21% CI [16% 25%]) patients before discharge (CDG 3b). The distributions vary between patient groups and depend also on the preoperative state of the patient, which is not considered here.

The median KPS scale at discharge was significantly lower for patients with complications (p = 5e-22, Mann-Whitney U-test). The median KPS at discharge without complication and for different CDG is shown in Fig 3B. The KPS scale and the CDG grade were correlated with Spearman’s ρ = -0.3 (p = 3e-24). The median KPS was significantly lower for CDG grades 2, 3b, 4a and 5 (Mann-Whitney U-test after Bonferroni correction for multiple testing). The linear fit gave a slope of -6 KPS percentage points per increment of CDG.

The median postoperative length of hospitalisation was significantly higher in cases with a complication (10 vs. 5 days, p = 2e-34, Mann-Whitney U-test. The length of stay and the CDG grade were correlated with Spearman’s ρ = 0.4 (p = 2e-35). The linear fit gave a slope of 1.5 days per increment of CDG (Fig 3C).

Fig 3D compares the distributions of KPS in dCRF with and without complication. The prevalence of complications was significantly higher for KPSS < 80 (p = 0.033, Mann-Whitney U-test).

## 4. Discussion

### 4.1. Summary

In our intervention we prospectively register an entire population of patients with respect to the neurosurgical treatment provided, its clinical outcome and concurrent complications. We assure the completeness and accuracy of the data at several levels. Across all surgeries, we found a clear correlation between the severity of a complication at discharge and patient outcome. Discussing the aggregated data among physicians on a monthly basis has a direct impact on patient treatment.

### 4.2. Interpretation

To our knowledge, we present here the first comprehensive patient registry focused on cranial neurosurgery in a German-speaking country. Monitoring the quality of medical treatment is an important issue both nationally [2] and internationally [1]. Other registries comprise only subsets of neurosurgical patients, e.g. spinal patients [16], neurovascular patients [18], or tumour patients [17]. The design of our registry was inspired by the Dutch neurosurgery registry [22], a widespread registry in general surgery [9–12], and a national registry for general surgery [15]. We aimed at a transparent design of the registry so that it may possibly serve as starting point for a national patient registry for benchmarking between different neurosurgery departments. Our quest for outcome transparency is documented in the yearly quality management report of the hospital and is thus seen as a contribution to sharpen the profile of our hospital as a high-quality health care provider.

As to rating complications, the severity grading enhances the willingness to register also complications that are minor in the view of the physician. Also minor complications (CDG 1 and CDG 2) can have a clear impact on outcome (Fig 3B) and on treatment costs [23]. We are aware that a number of grading schemes have been proposed explicitly for neurosurgery [6–8]. We have decided to use the Clavien Dindo classification system (CDG, Table 1), which has gained wide acceptance with more than 3000 citations and is used worldwide in many settings including large databases and randomized controlled trials [9–12]. Overall, the CDG correlates reasonably well with both outcome (Fig 3B) and length of stay (Fig 3C), mainly due to the large number of cases with CDG 1 and CDG 2. Each complication was additionally classified as either medical or surgical [24]. For data acquisition, the Landriel scheme [8] might be more intuitive, because any complication without invasive treatment is class 1, whereas it may be CDG 1 or CDG 2. However, as seen in the direct comparison (Table 1), the two schemes can be easily mapped onto each other so that statistical analyses can be easily compared.

The Buenos Aires study registered 167 complications in 1190 patients (14% CI [12% 16%]) but does not report clinical outcome [8]. The Milano study registered 228 complications in 1008 patients (23% CI [20% 25%]) but reports outcome only for patients with complications [25]. Our rate of 24% is in the same range. In general, we found a clear correlation between the severity of a complication and clinical outcome and length of hospital stay (Fig 3B and 3C). Interestingly, the median outcome for CDG 3 (complication requiring surgical intervention) was not worse than for CDG 1 or CDG 2, similar as in Milano [25]. Table 1 shows that the severity grading of our complications was very similar to that of Milano, while in Buenos Aires more life threatening complications were registered. Patient death was registered more frequently in our cohort, probably because we classified death of any reason as a complication if death occurred within 30 days of the surgery. Differences between cohorts may be due, apart from treatment quality, to the rigour of registration, the characteristics of the patient population and category/type and complexity of surgical procedures.

### 4.3. Limitations and outlook

Complications are graded by their severity in a therapy-oriented complication score system [12]. While this approach has many advantages, some complications are classified as low grade, while they may have a severe impact on the patient. This is particularly true for new neurological deficits that cannot be treated. Their impact is measured by the outcome scales in our registry. Outcome grading and complication severity grading thus serve a complimentary purpose. In future work we will investigate the correlation between CDG and KPS in selected subpopulations of patients.

The limited personal resources at our institution make the quest for data completeness and data accuracy a continuous challenge. This is a general issue in recording complications [26, 27]. We see rapid and frequent feedback of aggregated data (S1 Table) as an important aspect to motivate the colleagues in charge of filling out the forms in parallel to their clinical duties.

As a next step, we will move away from paper CRFs and filemaker^®^ to integrate the data acquisition and storage into the electronic patient record system (KIS). This will help to assure data completeness and safety and remove redundancies, e.g. with patient names, treatment times and some clinical scales. The following obstacles have to be overcome: 1) CRFs have to be optimized to a final version. 2) The graphical appeal of CRFs has to be compensated by KIS accessibility. 3) Accomplishing an interface to KIS has to be outsourced to the provider (www.cistec.com). 4) The data has to be extracted from KIS and analysed in a separate program, where general data mining solutions on the basis of Microsoft Amalga^®^ are currently being discussed.

Further, we are currently working to obtain the ISO 9001 certificate to improve the patient registry with external audits.

## 5. Conclusions

The registry serves to streamline and to complete information flow in the clinic, to identify complication rates and trends early for the internal quality monitoring. This endeavour needs a sustained long-term commitment. The impact of complications can be studied in closely defined patient groups. While the therapy-oriented complication scores correlate reasonably well with outcome and length of stay, they do not account for new deficits that cannot be treated. Outcome grading and complication severity grading thus serve a complimentary purpose. Overall, the obtained data support the communication within the clinic, with external partners, and with patients. Conversely, the registry influences clinical practice in that it demands rigorous documentation and standard operating procedures. We aimed at a transparent design of the registry so that it may possibly serve as starting point for a national patient registry for benchmarking between different neurosurgery departments.

## Supporting Information

S1 Forms Case report forms (CRF). Admission aCRF, Surgery sCRF, Discharge dCRF, Follow-up fCRF.
(PDF)

S1 Table Example listing of patients used in the monthly staff discussion of complications (November 2015). Alter—age; Teilsumme—subtotal; Eingriff—Intervention; Anz. Operationen—number of surgeries on this patient; OP wegen Kompl—surgery due to complication; Liegedauer postop—length of stay in hospital after surgery; neues neurology Defiz—new neurological deficit; Epianfall erstmalig—first time epileptic seizure; Nachblutung—recurrent bleeding; Verstorben < 30d —death within 30 days after surgery; andere Komplik—other complication; Harnwegsinfekt—urinary tract infection; Anzahl pro Austritt—number of complications noted at discharge; Aufenthalt nach Austritt—place of residence after discharge; erster Operateur (Fall)—surgeon of first intervention; Knocheneingriff—skull treatment; Externe—external ventricular drain; Alltagsumfeld—home; eigenes Spital / andere—same hospital different clinic.
(PDF)
