# Supplementary material for: A Patient Registry to Improve Patient Safety: Recording General Neurosurgery Complications
Source: PLoS One. 2016 Sep 26;11(9):e0163154. doi: 10.1371/journal.pone.0163154 (PMC5036891; doi:10.1371/journal.pone.0163154)
Supplement: S1 Forms — Admission aCRF, Surgery sCRF, Discharge dCRF, Follow-up fCRF. (PDF) [file pone.0163154.s001.pdf]

UniversitätsSpital Zürich

Klinik für Neurochirurgie
Frauenklinikstrasse 10
CH-8091 Zürich

Klinikdirektor
Prof. Dr. med. L. Regli

(ggf. Patientenkleber)

Name, Vorname:

Geburtsdatum:

Patientennummer:

Eintritt

Zuständiger Assistenzarzt:

Zuständiger Oberarzt:

Eintrittsdatum:

Station: ☐ M ☐ IPS ☐ N ☐ andere

☐ **Keine invasive Therapie erfolgt** *(keine Operation, keine Intervention, keine ELD etc. In dem Fall kein Ausfüllen der Scores unten erforderlich)*

☐ **Patient intubiert eingeliefert** *(In dem Fall kein Ausfüllen der Scores unten erforderlich, nur umseitig die Fragen an den Patienten)*

Eintrittsdaten

Eintrittsart (PRISMA)

- ☐ Notfall
- ☐ angemeldet, geplant
- ☐ Geburt
- ☐ interner Übertritt mit Wechsel des Adminfalls
- ☐ Verlegung innerhalb von 24 Stunden
- ☐ anderes
- ☐ unbekannt

Aufenthalt vor Eintritt (PRISMA)

- ☐ Alltagsumfeld (Privathaushalt o.Ä.)
- ☐ zu Hause mit Spitex-Versorgung
- ☐ Krankenheim, Pflegeheim
- ☐ Altersheim, sozialmedizin. Institution
- ☐ Psychatrische Klinik
- ☐ Rehabilitationsklinik
- ☐ anderes Krankenhaus
- ☐ andere
- ☐ unbekannt

Funktion im Alltag

Karnofsky Index

- ☐ 100 keine Beschwerden
- ☐ 90 fähig zu normaler Aktivität, geringe Symptome
- ☐ 80 normale Aktivität, deutliche Symptome
- ☐ 70 Selbstversorgung; Arbeit unmöglich
- ☐ 60 Selbständig in meisten Bereichen
- ☐ 50 Hilfe wird oft in Anspruch genommen
- ☐ 40 behindert, qualifizierte Hilfe nötig
- ☐ 30 Schwerbehinderung, Hospitalisation erforderlich
- ☐ 20 Schwer krank, Intensivmedizinische Massnahmen
- ☐ 10 moribunder Patient
- ☐ 0 Tod

Glasgow Coma Scale

Motorische Antwort

- ☐ 6 Befolgt Aufforderungen
- ☐ 5 Gezielte Abwehr
- ☐ 4 Ungezielte Abwehr
- ☐ 3 Beugeabwehr
- ☐ 2 Strecksynergismen
- ☐ 1 keine Reaktion

Augen öffnen

- ☐ 4 spontan offen
- ☐ 3 auf Aufforderung
- ☐ 2 auf Schmerz
- ☐ 1 keine Reaktion

Verbale Antwort

- ☐ 5 orientiert
- ☐ 4 desorientiert
- ☐ 3 unzusammenhängend
- ☐ 2 Laute
- ☐ 1 keine Reaktion

Modified Rankin Scale (mRS)

- ☐ 0 keine Symptome
- ☐ 1 keine nennenswerten Beschwerden, norm. Aktivität
- ☐ 2 Leichte Behinderung, Selbstversorgung
- ☐ 3 Mittelgradige Behinderung, wenig Hilfe, läuft selbst
- ☐ 4 Mittelschwere Behinderung, Hilfe bei Pflege und Gehen
- ☐ 5 Schwere Behinderung, Bettlägerigkeit, Inkontinenz
- ☐ 6 Tod

Neuropsychologie

Funktion kognitiv

- ☐ Verhalten adäquat
- ☐ Auffälliges Verhalten
- ☐ unbekannt

Montreal Cognitive Assessment (max. 30 Punkte)

- ☐ Nicht erhoben

NIH Stroke Scale ☐ unauffällig (NIHSS = 0)

Item	Punkte	Beschreibung
Bewusstsein	0	Wach
	1	Somnolent, erweckbar
	2	Stuporös
	3	Komatös
Orientierung (Alter, Monat)	0	Beide Antworten richtig
	1	Eine Antwort richtig
	2	Keine Antwort richtig
Auf-forderungen (2 Stück)	0	Beide korrekt erfüllt
	1	Eine korrekt befolgt
	2	Keine korrekt befolgt
Blickwendung	0	Normal
	1	Partielle Parese
	2	Forcierte Deviation
Gesichtsfeld	0	Normal
	1	Partielle Hemianopsie
	2	Komplette Hemianopsie
	3	Bilaterale Hemianopsie/ Amaurose
Mimik	0	Normal
	1	Geringe Asymmetrie
	2	Partielle Facialisparese
	3	Facialis-Plegie
Armmotorik (L / R)	0 0	M5
	1 1	M4, Abs. innert 10s
	2 2	M3
	3 3	M2 oder M1
	4 4	Plegie
Beinmotorik (L / R)	0 0	M5
	1 1	M4, Abs. innert 10s
	2 2	M3
	3 3	M2 oder M1
	4 4	Plegie
Ataxie	0	Normal
	1	In einer Extremität
	2	In zwei oder mehr Extr.
Sensibilität	0	Normal
	1	Partieller Verlust
	2	Schwerer bis vollständiger Verlust
Sprache	0	Normal
	1	Leichte Aphasie
	2	Schwere Aphasie
	3	Globale Aphasie/ Stumm
Dysarthrie	0	Normal
	1	Verwaschen, verständl.
	2	Unverständlich/Stumm
Neglect	0	Normal
	1	Partieller halbseitiger Neglect (unimodal)
	2	Kompletter halbseitiger Neglect (multimodal)

Komplikationen

Führten Komplikationen zum Eintritt?
- Ja
- Nein
- unbekannt

	CDG		Datum (ddmmyy)	
Wunddehiszenz (T81.3)				
Wundinfekt (T81.4)				
Nachblutung (T81.0)				
Liquorfistel (G97.80)				
Ventrikulitis (G04.9)				
Meningitis (G00)				
Cerebraler Infarkt (I63)				
Shuntproblem				
Epilepsie erstmalig (G40)				
Thrombose (I80)				
Lungenembolie (I26)				
Pneumonie (J18)				
Harnwegsinfekt (N39)				

Clavien-Dindo-Grade (CDG)

1	Any deviation from normal postoperative course
2	Requiring pharmacological treatment
3a	Requiring surgical intervention without general anaesthesia
3b	Requiring surgical intervention with general anaesthesia
4a	Life-threatening complication, ICU, single-organ dysfunction
4b	Life-threatening complication, ICU, multi-organ dysfunction
5	Death of patient

Surgical Site Infection

Superficial	Ja	Nein
Deep	Ja	Nein
Organ space	Ja	Nein
Implant	Ja	Nein

Art der schwersten Komplikation

Expected	Ja	Nein
Related	Ja	Nein
Serious (disabling, life threatening)	Ja	Nein

Epilepsie

Epileptischer Anfall präoperativ?
- Ja
- Nein
- unbekannt

Soziales Umfeld und Ressourcen (Fragen an den Patienten)

Wohnsituation
- Einpersonenhaushalt
- Mehrpersonenhaushalt

Lebt im Haushalt eine Person, die sich um Sie kümmert?
- Ja
- Nein

Wie ist Ihre Gesundheit im Allgemeinen?
- Sehr gut
- gut
- mittelmässig
- schlecht
- sehr schlecht

Ausbildung
- keine abgeschlossene Schulbildung
- obligatorische Volksschule
- Berufslehre / Berufsschule
- Maturitätsschule
- Höhere Fach- oder Berufsschule
- Universität, Hochschule
- unbekannt

Rauchen Sie?
- ja, ____ Z/Tag
- nein, noch nie
- nein, sistiert vor ____ Jahren
- ____ py

Berufliche Situation derzeit
- erwerbstätig 100% (auch wenn arbeitsunfähig / krank)
- erwerbstätig Teilzeit (auch wenn arbeitsunfähig / krank)
- in Ausbildung / Umschulung
- Haushalt / Freiwilligentätigkeit
- keine Tätigkeit
- arbeitslos gemeldet
- IV-Bezüger (Invalidenrente)
- AHV-Bezüger (Altersrente)
- andere
- unbekannt

**Datum:**		**Kisim username Arzt**			
**Datum:**		**Visum Oberarzt**		**Visum Daten-erfasser**	

**Datum der OPERATION:**
**Operateur 1:**
**Operateur 2:**

PATIENT KLEBER GROSS oder

Name, Vorname

Geburtsdatum
(ggf. Patientennummer)

1. INDIKATION FÜR HEUTIGEN EINGRIFF (max. 3)
Cerebrovaskulär
□ Parenchymale Blutung (ICB, nicht Trauma)
□ Ischämie
□ Aneurysma Komplex □ nein □ ja*
□ Cavernom (CCM)
□ durale Fistel (DAVF)
□ Arteriovenöse Malformation (AVM)
□ andere (Freitext)
Lesion □ rupturiert □ nicht rupturiert
SAH: WFNS Grad □ 1 □ 2 □ 3 □ 4 □ 5
AVM: Spetzler-Martin Grad □ 1 □ 2 □ 3 □ 4 □ 5
* >25mm, Äste aus dem Sack (inkl. Perforatoren), vorherige Behandlung, auffällige Wand (thrombosiert, verkalkt, ateriosklerotisch).

Tumor zerebral
□ Low Grade Gliom
□ High Grade Gliom
□ Gliom (andere)
□ Metastase
□ Hypophysenadenom
□ Kraniopharyngeom
□ Meningeom
□ Schwannom Vestibularis
□ andere (Freitext)

□ **Komplikation** (bitte im Freitext genauer beschreiben)

Trauma
□ Hämatom epidural
□ Hämatom subdural akut
□ Hämatom subdural chronisch
□ Hämatom parenchymal (nur Trauma)
□ andere (Freitext)

Liquorzirkulationsstörung
□ Hydrocephalus NPH
□ Hydrocephalus occlusivus
□ Hydrocephalus communicans/malresorptivus
□ Shunt Dysfunktion / Revision / Infekt
□ Rhinoliquorrhoe
□ andere (Freitext)

Spinal
□ Bandscheibenvorfall
□ Fehlstellung
□ Stenose
□ Zyste / Abszess / Tumor / Gefässmissbildung
□ Hämatom
□ Metastase
□ Syringo-/Hydromyelie
□ andere (Freitext)

Neurologische/ Psychiatrische Indikation
□ Bewegungsstörung
□ Epilepsie
□ Neuralgie
□ andere (Freitext)

ANDERE INDIKATION (Freitext inkl. Kategorie)

2. EINGRIFF (max. 3 Einträge)
Cerebrovaskulär
□ Clipping / Wrapping
□ Gefässverschluss (Trapping)
□ Revaskularisation (EC-IC / IC-IC Bypass)
□ Resektion/ Interruption Nidus AVM, DAVF, CCM
□ Carotis Endarterektomie

Tumor
□ Resektion / Biopsie (Ausmass Frage 5)

Spinal
□ Hemilaminektomie
□ Laminektomie
□ Fenestration
□ Anteriore Diskektomie mit/ohne Cage Implantation
□ Instrumentation / Stabilisierung
□ Infiltration
□ Foraminotomie

Neuromodulation / Epilepsie
□ Implantation Elektrode
□ Implantation Stimulator
□ VNS Implantation
□ SCS Implantation
□ Explantation Elektrode
□ Amygdala-Hippokampektomie (AHE)
□ Läsionektomie
□ Mikrovaskuläre Dekompression

Weitere Eingriffe
□ Transphenoidaler Eingriff

□ Shunt VP oder VA
□ Externe Ventrikeldrainage EVD
□ Ventrikulozisternostomie
□ ICP/ Neuromonitoring Sonde legen

□ Evakuation Kraniotomie
□ Evakuation Bohrloch
□ Dekompressive Kraniektomie

□ Knocheneingriff Palacos / PEEK / Knochendeckel
□ Wundrevision mit/ohne Knochenrevision

□ **Andere Eingriffe (Freitext)**

ANDERER EINGRIFF (Freitext inkl. Kategorie)

BITTE WENDEN!

3. LOKALISATION (max. 2 Einträge)

Vaskulär (Aneurysma Lokalisation)
□ ICA
□ ACA proximal / ACom
□ ACA distal
□ MCA proximal
□ MCA distal
□ Hinterer Kreislauf

Intraparenchymal (für Tumor und Vaskulär)
□ Zentralregion
□ Insula
□ Hemisphärisch andere
□ Stammganglien / Thalamus
□ Pinealis Region
□ Hypophyse
□ Hirnstamm
□ Kleinhirn
□ Intraventrikulär
□ Hippocampus / Amygdala

Extraparenchymal
□ Vordere Schädelgrube inkl. Nase, Orbita
□ Mittlere Schädelgrube
inkl. Sella, Sinus Cavernosus
□ Hintere Schädelgrube
inkl. Kraniozervikaler Übergang
□ Konvexität / Falx

Wirbelsäule (ankreuzen)

	zervikal	thorakal	lumbal	sakral
extradural				
intradural extra-m				
intradural intra-m				
intraossär				
Weichteil				

Sonstige
□ Körperstamm

4. KONTEXT (JE EINE ANGABE)
Erstoperation □ ja □ nein
Eingriff indiziert aufgrund einer Komplikation?
(neu auf Seite 1 angeben!)
Wurde der Operationstermin verschoben?
□ nein □ ja, medizinisch indiziert □ ja, organisatorisch

5. EINSCHÄTZUNG TUMOR-RESEKTION

Gliom	Gross total resection □ >98%	Subtotal resection □ >90%	Partial resection □ <90%	Biopsy □	
Metastase entfernung	Total □	Partiell □	Biopsie □		
Meningeom Simpson	□ 1	□ 2	□ 3	□ 4	□ 5

6. SCHNELLSCHNITT BEFUND

Visum **OPERATEUR** und Datum für Punkte 1-6:

7. INTRAOPERATIVES NEUROMONITORING
□ IONM
□ SEP
□ MEP
□ CoMEP
□ AEP
□ VEP
□ Hirnnerven/Einzelwurzel
□ Cortex-Stimulation
□ EEG

8. LAGERUNG
□ Bauchlage
□ Seitenlage
□ Halbseitenlage
□ Rückenlage
□ Sitzposition
□ Umlagerung

9. GERÄTE
□ Mikroskop
□ Navigation
□ Ultraschall
□ C-Bogen
□ Endoskop
□ Stereotaxie-Rahmen
□ iMRI 3T
□ 5-ALA
□ Laser ELANA
□ CT AIRO
□ Wach-OP
□ Flowmeter
□ ICG
□ Microdoppler

10. PROBENMATERIAL
□ Schnellschnitt
□ Neuropathologie
□ Forschungslabor
□ Bakteriologie
□ Zytologie
□ Neuroimmunologie
□ andere

11. OP-Saal
□ 1 □ 2 □ 3 □ NF-OPS
□ IPS □ ORL □ KISPI □andere

12. OP-Pflege

Instrumentierung	
Zudienung	

13. OP-ZEITEN
Übernahme OP :
OP Beginn :
OP Ende :
Übergabe OP :

14. Abrechnung
□ KISIM □ LEP

Visum **LEITSTELLE** und Datum für Punkte 8-14

Visum Datenerfasser / Visum Sponsor / Visum Bildarchiv

UniversitätsSpital Zürich

Klinik für Neurochirurgie
Frauenklinikstrasse 10
CH-8091 Zürich

Klinikdirektor
Prof. Dr. med. L. Regli

(ggf. Patientenkleber)

Name, Vorname:

Geburtsdatum:

Patientennummer:

Austritt

Zuständiger Assistenzarzt:

Zuständiger Oberarzt:

Austrittsdatum: **Operateur:** **Station:** ☐ M ☐ IPS ☐ N ☐ andere

Indikation für OP
☐ Vaskulär ☐ Tumor ☐ Trauma ☐ Liquor ☐ Spinal ☐ Komplikation ☐ andere

☐ **Keine invasive Therapie erfolgt**
(kein Ausfüllen der Scores erforderlich)

Austrittsdaten

Art des Austritts (AQC)
- ☐ geheilt
- ☐ gebessert
- ☐ nicht gebessert / unverändert
- ☐ verschlechtert
- ☐ nicht beurteilbar
- ☐ Exitus intraoperativ
- ☐ Exitus postoperativ
- ☐ Exitus ohne Zusammenhang mit OP
- ☐ Exitus innert 30 Tagen nach OP

Aufenthalt nach Austritt (PRISMA)
- ☐ Todesfall
- ☐ Alltagsumfeld (Privathaushalt o.Ä.)
- ☐ Krankenheim, Pflegeheim
- ☐ Altersheim, sozialmedizin. Institution
- ☐ Psychatrische Klinik
- ☐ Rehabilitationsklinik
- ☐ anderes Krankenhaus
- ☐ andere
- ☐ unbekannt
- ☐ eigenes Spital / andere Abteilung

Behandlung nach Austritt (PRISMA)
- ☐ Todesfall
- ☐ geheilt oder kein Handlungsbedarf
- ☐ ambulante Behandlung
- ☐ ambulante Pflege / Spitex
- ☐ stationäre Behandlung
- ☐ Rehabilitation
- ☐ andere
- ☐ unbekannt

Exitus Datum

Funktion im Alltag

Karnofsky Index
- ☐ 100 keine Beschwerden
- ☐ 90 fähig zu normaler Aktivität, geringe Symptome
- ☐ 80 normale Aktivität, deutliche Symptome
- ☐ 70 Selbstversorgung; Arbeit unmöglich
- ☐ 60 Selbständig in meisten Bereichen
- ☐ 50 Hilfe wird oft in Anspruch genommen
- ☐ 40 behindert, qualifizierte Hilfe nötig
- ☐ 30 Schwerbehinderung, Hospitalisation erforderlich
- ☐ 20 Schwer krank, Intensivmedizinische Massnahmen
- ☐ 10 moribunder Patient
- ☐ 0 Tod

Glasgow Outcome Score (bei Hirnschädigungen)
- ☐ 1 Tod
- ☐ 2 Persistierender vegetativer Zustand
- ☐ 3 Schwere Behinderung
- ☐ 4 Mässige Behinderung
- ☐ 5 Geringe Behinderung
- ☐ N/A

Modified Rankin Scale (mRS)
- ☐ 0 keine Symptome
- ☐ 1 keine nennenswerten Beschwerden, norm. Aktivität
- ☐ 2 Leichte Behinderung, Selbstversorgung
- ☐ 3 Mittelgradige Behinderung, wenig Hilfe, läuft selbst
- ☐ 4 Mittelschwere Behinderung, Hilfe bei Pflege und Gehen
- ☐ 5 Schwere Behinderung, Bettlägerigkeit, Inkontinenz
- ☐ 6 Tod

Neuropsychologie

Funktion kognitiv
- ☐ Verhalten adäquat
- ☐ Auffälliges Verhalten
- ☐ unbekannt

Montreal Cognitive Assessment (max. 30 Punkte)
- ☐ Nicht erhoben

NIH Stroke Scale ☐ unauffällig (NIHSS = 0)

Bewusstsein
- 0 Wach
- 1 Somnolent, erweckbar
- 2 Stuporös
- 3 Komatös

Orientierung (Alter, Monat)
- 0 Beide Antworten richtig
- 1 Eine Antwort richtig
- 2 Keine Antwort richtig

Auf-forderungen (2 Stück)
- 0 Beide korrekt erfüllt
- 1 Eine korrekt befolgt
- 2 Keine korrekt befolgt

Blickwendung
- 0 Normal
- 1 Partielle Parese
- 2 Forcierte Deviation

Gesichtsfeld
- 0 Normal
- 1 Partielle Hemianopsie
- 2 Komplette Hemianopsie
- 3 Bilaterale Hemianopsie/ Amaurose

Mimik
- 0 Normal
- 1 Geringe Asymmetrie
- 2 Partielle Facialisparese
- 3 Facialis-Plegie

Armmotorik (L / R)
- 0 0 M5
- 1 1 M4, Abs. innert 10s
- 2 2 M3
- 3 3 M2 oder M1
- 4 4 Plegie

Beinmotorik (L / R)
- 0 0 M5
- 1 1 M4, Abs. innert 10s
- 2 2 M3
- 3 3 M2 oder M1
- 4 4 Plegie

Ataxie
- 0 Normal
- 1 In einer Extremität
- 2 In zwei oder mehr Extr.

Sensibilität
- 0 Normal
- 1 Partieller Verlust
- 2 Schwerer bis vollständiger Verlust

Sprache
- 0 Normal
- 1 Leichte Aphasie
- 2 Schwere Aphasie
- 3 Globale Aphasie/ Stumm

Dysarthrie
- 0 Normal
- 1 Verwaschen, verständl.
- 2 Unverständlich/Stumm

Neglect
- 0 Normal
- 1 Partieller halbseitiger Neglect (unimodal)
- 2 Kompletter halbseitiger Neglect (multimodal)

Histopathologie

Austritt

Neuroepitheliale Tumore
- Astrocytom Grad II
- Anaplastisches Astrozytom Grad III
- Glioblastom
- Gliosarkom
- Pilozytisches Astrocytom (WHO Grad I)
- Pleomorphes Xanthoastrocytom
- Subependymales Riesenzellastrozytom
- Oligodendrogliom (WHO Grad II und III)
- Ependymom
- Anapastisches Ependymom
- Oligoastrozytom
- Plexuspapillom
- Gangliocytom
- Pineocytom
- Pineoblastom
- Neuroblastom
- PNET (incl. Medulloblastom)

Meningeale Tumore
- Meningeom (WHO Grad I)
- Atypisches Meningeom (WHO Grad II)
- Anaplastisches Meningeom (WHO Grad III)

Tumore von Hirn- und Spinalnerven
- Schwannom
- Neurofibrom
- Maligner peripherer Nervenscheidentumor

Hämatopoetische Neoplasien
- Lymphom
- Plasmozytom

Stammzelltumore
- Germinom
- Embryonales Carcinom
- Coriocarcinom
- Teratom

Cystische Tumore
- Rathke'sche Tasche Cyste
- Epidermoidcyste
- Dermoidcyste
- Kolloidcyste

Selläre Tumore
- Hypophysenadenom
- Kraniopharyngeom

Metastatische Tumore
- Metastase

- Andere:

Bildgebung und Diagnose

Zerebrovaskulär

Aktuelle Bildgebung vorhanden? Ja Nein unbekannt
Wenn Ja, welche: CT MRI DSA

Aneuysma
Komplette Occlusion/Resektion Ja Nein unbekannt
Bypass durchgängig Ja Nein unbekannt

AVM/dAVF/Cavernom
Komplette Occlusion/Resektion Ja Nein unbekannt

Stroke
Neue Ischämie Ja Nein unbekannt
Bypass durchgängig Ja Nein unbekannt
Wiedereröffnung Gefäss komplett inkomp unbekannt

Tumor

Postop. CT/MRI vorhanden? Ja Nein unbekannt
In Tumorboard besprochen? Ja Nein unbekannt

Tumorentfernung retrospektiv (anhand postop. CT/MRI)

Gliom
- Complete resection
- GTR (>98%)
- Subtotal resection (>90%)
- Partial resection (< 90%)
- Biopsy

Datum der aktuellen Bildgebung:

Metastase / andere Tumore
- Totalentfernung
- Partielle Entfernung
- Biopsie

Komplikationen

Gab es Komplikationen im peri-/postoperativen Verlauf?
- Ja
- Nein
- unbekannt

	CDG	d	Datum (ddmmyy)	Zeitraum
Wunddehiszenz (T81.3)				
Wundinfekt (T81.4)				
Nachblutung (T81.0)				
Liquorfistel (G97.80)				
Ventrikulitis (G04.9)				
Meningitis (G00)				
Cerebraler Infarkt (I63)				
Shuntproblem				
neues neurologisches Defizit				
Epilepsie erstmalig (G40)				
Verstorben < 30 Tage post OP				
Thrombose (I80)				
Lungenembolie (I26)				
Pneumonie (J18)				
Harnwegsinfekt (N39)				

Komplikations-Zeitraum:
1 **intraoperativ**
2 **direkt postoperativ (innert 48 h)**
3 **in ersten 18 Tagen postoperativ**
4 **bis zur ersten postoperativen Kontrolle (idR 6 Wo)**
5 **im Langzeitverlauf**

Clavien-Dindo-Grade (CDG)
1 Any deviation from normal postoperative course
2 Requiring pharmacological treatment
3a Requiring surgical intervention without general anaesthesia
3b Requiring surgical intervention with general anaesthesia
4a Life-threatening complication, ICU, single-organ dysfunction
4b Life-threatening complication, ICU, multi-organ dysfunction
5 Death of patient

d Zusatzcode für Defizit zum Beurteilungszeitpunkt (auch transient)

Surgical Site Infection
Superficial Ja Nein
Deep Ja Nein
Organ space Ja Nein
Implant Ja Nein

Epileptischer Anfall postoperativ?
- Ja
- Nein
- unbekannt

Art der schwersten Komplikation
Expected Ja Nein
Related Ja Nein
Serious Ja Nein
(disabling / life threatening / prolonging hospitalisation)

Datum: | **Kisim username Arzt**
Datum: | **Visum Oberarzt** | **Visum Daten-erfasser**

UniversitätsSpital Zürich
Klinik für Neurochirurgie
Frauenklinikstrasse 10
CH-8091 Zürich

Klinikdirektor
Prof. Dr. med. L. Regli

(ggf. Patientenkleber)

Name, Vorname:

Geburtsdatum:

Patientennummer:

Sprechstunde

**Sprechstunde Datum:**		**Behandelnder Arzt:**		**Patient erschienen:**	☐ Ja ☐ Nein
**Indikation:**		**Procedere:**		**Monate postop.:**	

Lost to follow-up ☐

Zustand derzeit

Zustand derzeit (AQC)
- ☐ geheilt
- ☐ gebessert
- ☐ nicht gebessert / unverändert
- ☐ verschlechtert
- ☐ nicht beurteilbar
- ☐ Exitus intraoperativ
- ☐ Exitus postoperativ
- ☐ Exitus ohne Zusammenhang mit OP
- ☐ Exitus innert 30 Tagen nach OP

Aufenthalt derzeit (PRISMA)
- ☐ Todesfall
- ☐ Alltagsumfeld (Privathaushalt o.Ä.)
- ☐ Krankenheim, Pflegeheim
- ☐ Altersheim, sozialmedizin. Institution
- ☐ Psychatrische Klinik
- ☐ Rehabilitationsklinik
- ☐ anderes Krankenhaus
- ☐ andere
- ☐ unbekannt
- ☐ eigenes Spital / andere Abteilung

Behandlung derzeit (PRISMA)
- ☐ Todesfall
- ☐ geheilt oder kein Handlungsbedarf
- ☐ ambulante Behandlung
- ☐ ambulante Pflege / Spitex
- ☐ stationäre Behandlung
- ☐ Rehabilitation
- ☐ andere
- ☐ unbekannt

Exitus Datum ____________

Funktion im Alltag

Karnofsky Index
- ☐ 100 keine Beschwerden
- ☐ 90 fähig zu normaler Aktivität, geringe Symptome
- ☐ 80 normale Aktivität, deutliche Symptome
- ☐ 70 Selbstversorgung; Arbeit unmöglich
- ☐ 60 Selbständig in meisten Bereichen
- ☐ 50 Hilfe wird oft in Anspruch genommen
- ☐ 40 behindert, qualifizierte Hilfe nötig
- ☐ 30 Schwerbehinderung, Hospitalisation erforderlich
- ☐ 20 moribunder Patient
- ☐ 10 Tod

Glasgow Outcome Score (bei Hirnschädigungen)
- ☐ 1 Tod
- ☐ 2 Persistierender vegetativer Zustand
- ☐ 3 Schwere Behinderung
- ☐ 4 Mässige Behinderung
- ☐ 5 Geringe Behinderung
- ☐ N/A

Modified Rankin Scale (mRS)
- ☐ 0 keine Symptome
- ☐ 1 keine nennenswerten Beschwerden, norm. Aktivität
- ☐ 2 Leichte Behinderung, Selbstversorgung
- ☐ 3 Mittelgradige Behinderung, wenig Hilfe, läuft selbst
- ☐ 4 Mittelschwere Behinderung, Hilfe bei Pflege und Gehen
- ☐ 5 Schwere Behinderung, Bettlägerigkeit, Inkontinenz
- ☐ 6 Tod

Neuropsychologie

Funktion kognitiv
- ☐ Verhalten adäquat
- ☐ Auffälliges Verhalten
- ☐ unbekannt

Mini Mental State (fakultativ)
(uneingeschränkt: 30 Punkte): ____

- ☐ Nicht erhoben

NIH Stroke Scale ☐ unauffällig (NIHSS = 0)

Item	Score
Bewusstsein	0 Wach; 1 Somnolent, erweckbar; 2 Stuporös; 3 Komatös
Orientierung (Alter, Monat)	0 Beide Antworten richtig; 1 Eine Antwort richtig; 2 Keine Antwort richtig
Auf-forderungen (2 Stück)	0 Beide korrekt erfüllt; 1 Eine korrekt befolgt; 2 Keine korrekt befolgt
Blickwendung	0 Normal; 1 Partielle Parese; 2 Forcierte Deviation
Gesichtsfeld	0 Normal; 1 Partielle Hemianopsie; 2 Komplette Hemianopsie; 3 Bilaterale Hemianopsie/ Amaurose
Mimik	0 Normal; 1 Geringe Asymmetrie; 2 Partielle Facialisparese; 3 Facialis-Plegie
Armmotorik (L / R)	0 0 M5; 1 1 M4, Abs. innert 10s; 2 2 M3; 3 3 M2 oder M1; 4 4 Plegie
Beinmotorik (L / R)	0 0 M5; 1 1 M4, Abs. innert 10s; 2 2 M3; 3 3 M2 oder M1; 4 4 Plegie
Ataxie	0 Normal; 1 In einer Extremität; 2 In zwei oder mehr Extr.
Sensibilität	0 Normal; 1 Partieller Verlust; 2 Schwerer bis vollständiger Verlust
Sprache	0 Normal; 1 Leichte Aphasie; 2 Schwere Aphasie; 3 Globale Aphasie/ Stumm
Dysarthrie	0 Normal; 1 Verwaschen, verständl.; 2 Unverständlich/Stumm
Neglect	0 Normal; 1 Partieller halbseitiger Neglect (unimodal); 2 Kompletter halbseitiger Neglect (multimodal)

Bildgebung und Diagnose

Tumor

Aktuelle Bildgebung vorhanden ☐ Ja ☐ Nein

Tumor Progress / Rezidiv: ☐ Ja ☐ Nein
falls ja: ☐ lokal ☐ nicht lokal ☐ lokal und nichtlokal

Zerebrovaskulär

Aktuelle Bildgebung vorhanden ☐ Ja ☐ Nein

Aneuysma

Komplette Occlusion/Resektion	☐ Ja	☐ Nein	☐ unbekannt
Bypass durchgängig	☐ Ja	☐ Nein	☐ unbekannt
de novo Aneurysmata	☐ Ja	☐ Nein	☐ unbekannt
Vergrösserung andere Aneurys.	☐ Ja	☐ Nein	☐ unbekannt

AVM/dAVF/Cavernom

Komplette Occlusion/Resektion	☐ Ja	☐ Nein	☐ unbekannt

Stroke

Neue Ischämie	☐ Ja	☐ Nein	☐ unbekannt
CEA: Re-stenose	☐ Ja	☐ Nein	☐ unbekannt
Bypass durchgängig	☐ Ja	☐ Nein	☐ unbekannt

Sprechstunde

Komplikationen

Gab es Komplikationen im peri-/postoperativen Verlauf?
- ☐ Ja
- ☐ Nein
- ☐ unbekannt

	CDG	d	Datum (ddmmyy)
☐ Wunddehiszenz (T81.3)			
☐ Wundinfekt (T81.4)			
☐ Nachblutung (T81.0)			
☐ Liquorfistel (G97.80)			
☐ Ventrikulitis (G04.9)			
☐ Meningitis (G00)			
☐ Cerebraler Infarkt (I63)			
☐ Shuntproblem			
☐ neues neurologisches Defizit			
☐ Epilepsie erstmalig (G40)			
☐ Verstorben < 30 Tage post OP			
☐ Thrombose (I80)			
☐ Lungenembolie (I26)			
☐ Pneumonie (J18)			
☐ Harnwegsinfekt (N39)			
☐			

Clavien-Dindo-Grade (CDG)

1	Any deviation from normal postoperative course
2	Requiring pharmacological treatment
3a	Requiring surgical intervention without general anaesthesia
3b	Requiring surgical intervention with general anaesthesia
4a	Life-threatening complication, ICU, single-organ dysfunction
4b	Life-threatening complication, ICU, multi-organ dysfunction
5	Death of patient
d	Zusatzcode für Defizit zum Beurteilungszeitpunkt (auch transient)

Epileptischer Anfall seit letzter Visite?
- ☐ Ja
- ☐ Nein
- ☐ Unbekannt

Surgical Site Infection

Superficial	☐ Ja	☐ Nein
Deep	☐ Ja	☐ Nein
Organ space	☐ Ja	☐ Nein
Implant	☐ Ja	☐ Nein

Art der schwersten Komplikation

Expected	☐ Ja	☐ Nein
Related	☐ Ja	☐ Nein
Serious	☐ Ja	☐ Nein

(disabling / life threatening / prolonging hospitalisation)

Soziales Umfeld und Ressourcen (Fragen an den Patienten)

Wie ist Ihre Gesundheit im Allgemeinen?
- ☐ Sehr gut
- ☐ gut
- ☐ mittelmässig
- ☐ schlecht
- ☐ sehr schlecht

Rauchen Sie?
- ☐ ja, ____ Z/Tag
- ☐ nein, noch nie
- ☐ nein, sistiert vor ____ Jahren
- ____ py

Sind Sie mit Ihrer Narbe zufrieden?
- ☐ ja
- ☐ nein

Berufliche Situation derzeit
- ☐ erwerbstätig 100% (auch wenn arbeitsunfähig / krank)
- ☐ erwerbstätig Teilzeit (auch wenn arbeitsunfähig / krank)
- ☐ in Ausbildung / Umschulung
- ☐ Haushalt / Freiwilligentätigkeit
- ☐ keine Tätigkeit
- ☐ arbeitslos gemeldet
- ☐ IV-Bezüger (Invalidenrente)
- ☐ AHV-Bezüger (Altersrente)
- ☐ andere
- ☐ unbekannt

Datum: ____ **Kisim username Arzt** ____ **Visum Datenerfasser** ____
