# Supplementary material for: A Patient Registry to Improve Patient Safety: Recording General Neurosurgery Complications
Source: PLoS One. 2016 Sep 26;11(9):e0163154. doi: 10.1371/journal.pone.0163154 (PMC5036891; doi:10.1371/journal.pone.0163154)
Supplement: S1 Table — Alter—age; Teilsumme—subtotal; Eingriff—Intervention; Anz. Operationen—number of surgeries on this patient; OP wegen Kompl—surgery due to complication; Liegedauer postop—length of stay in hospital after surgery; neues neurology Defiz—new neurological deficit; Epianfall erstmalig—first time epileptic seizure; Nachblutung—recurrent bleeding; Verstorben < 30d —death within 30 days after surgery; andere Komplik—other complication; Harnwegsinfekt—urinary tract infection; Anzahl pro Austritt—number of complications noted at discharge; Aufenthalt nach Austritt—place of residence after discharge; erster Operateur (Fall)—surgeon of first intervention; Knocheneingriff—skull treatment; Externe—external ventricular drain; Alltagsumfeld—home; eigenes Spital / andere—same hospital different clinic. (PDF) [file pone.0163154.s002.pdf]

01.11.2015 .. 30.11.2015

Name	Alter	Indikation	Teilsumme	Eingriff	Anz. Operationen	OP wegen Kompl	Liegedauer postop	KPS ein	KPS aus	chirurgisch: neues neurolog Defiz.	Epianfall erstmalig	Nachblutung	Infarkt cerebri	Meningitis	Wundinfekt	Ventrikulitis	Verstorben <30d	andere Komplik.	medizinisch: Harnwegsinfekt	Thrombose	Liquorfistel	Lungenembolie	Pneumonie	Anzahl pro Austritt	3 CDG worst	4% Aufenthalt nach Austritt	erster Operateur (Fall)
										6	1	0	1	0	2	0	0	3	1	0	0	1	1				
B	72	andere		ICP/ Neuromonitoring	3	nein	35	30	40									ja						1	2	Rehabilitationsklinik	Sp
			1																								
G	52	Komplikation		Revaskularisation (EC-IC	2	ja	10	90	90	ja														1	3b	Rehabilitationsklinik	Es
P	36	Komplikation		Knocheneingriff Palacos	3	ja	5	60	70		ja													1	2	Alltagsumfeld	St
			2																								
S	57	Liquor		Externe	3	nein	12	40	40	ja					ja									4	3b	Rehabilitationsklinik	Sp
			1																								
S	68	Spinal		Instrumentation /	3		12	80	70													ja		1	2	Rehabilitationsklinik	Bu
			1																								
B	39	Tumor		Tumor Resektion/Biopsie	1	nein	9	90	90	ja														1	2	Alltagsumfeld	Pl
B	59	Tumor		Tumor Resektion/Biopsie	1	nein	10	90	80	ja														1	2	Alltagsumfeld	Pr
C	68	Tumor		Tumor Resektion/Biopsie	3	nein	28	80	80						ja									1	3b	Rehabilitationsklinik	Pl
G	69	Tumor		Tumor Resektion/Biopsie	2	nein	4	70	70				ja											1	1	Alltagsumfeld	Es
G	37	Tumor		Tumor Resektion/Biopsie	1	nein	4	90	70	ja														1	1	Alltagsumfeld	Bu
D	75	Tumor		Tumor Resektion/Biopsie	1	nein	16	60	70									ja						1	2	eigenes Spital / andere	Bu
Tl	49	Tumor		Tumor Resektion/Biopsie	2	nein	13	80	60	ja														1	1	Rehabilitationsklinik	Pl
A	27	Tumor		Tumor Resektion/Biopsie	4	nein	7	50	50														ja	1	2	Rehabilitationsklinik	Pl
			8																								
B	48	Vaskulär		Clipping / Wrapping	2	nein	17	50	90									ja	ja					2	2	Rehabilitationsklinik	Bu
			1																								
